# Applying Evidence-Centered Design to Measure Psychological Resilience: The Development and Preliminary Validation of a Novel Simulation-Based Assessment Methodology

**DOI:** 10.3389/fpsyg.2021.717568

**Published:** 2022-01-10

**Authors:** Sabina Kleitman, Simon A. Jackson, Lisa M. Zhang, Matthew D. Blanchard, Nikzad B. Rizvandi, Eugene Aidman

**Affiliations:** ^1^School of Psychology, The University of Sydney, Sydney, NSW, Australia; ^2^Centre for Translational Data Science, The University of Sydney, Sydney, NSW, Australia; ^3^Land Division, Defence Science and Technology Group, Edinburgh, SA, Australia

**Keywords:** assessment, simulation, resilience, evidence-centered design, machine learning

## Abstract

Modern technologies have enabled the development of dynamic game- and simulation-based assessments to measure psychological constructs. This has highlighted their potential for supplementing other assessment modalities, such as self-report. This study describes the development, design, and preliminary validation of a simulation-based assessment methodology to measure psychological resilience—an important construct for multiple life domains. The design was guided by theories of resilience, and principles of evidence-centered design and stealth assessment. The system analyzed log files from a simulated task to derive individual trajectories in response to stressors. Using slope analyses, these trajectories were indicative of four types of responses to stressors: thriving, recovery, surviving, and succumbing. Using Machine Learning, the trajectories were predictive of self-reported resilience (Connor-Davidson Resilience Scale) with high accuracy, supporting construct validity of the simulation-based assessment. These findings add to the growing evidence supporting the utility of gamified assessment of psychological constructs. Importantly, these findings address theoretical debates about the construct of resilience, adding to its theory, supporting the combination of the “trait” and “process” approaches to its operationalization.

## Introduction

Stress and adversity are an inevitable part of the human experience. However, not everyone is equally successful at overcoming potentially negative events. The construct of resilience offers one explanation for this. While there is no general agreement on what constitutes the psychological resilience, most researchers agree that it entails two core concepts: (1) the presence of a potential stressor, and (2) positive adaptation (see Fletcher and Sarkar, 2013 for a review). There is a growing body of research investigating different trajectories or divergent pathways of adjustment in response to acute and chronic stressors (Bonanno and Diminich, 2013). These trajectories can include thriving, recovery, surviving, and succumbing (O’Leary and Ickovics, 1995; Carver, 1998). However, little empirical evidence exists for these trajectories in response to a clearly referenced, acute stressor. Such research, however, can provide evidence for the positive adaptation, the key process suggested to underlie mental resilience (see Fletcher and Sarkar, 2013 for a review).

Well-designed interactive technology (e.g., games, simulations), also referred to as Virtual Performance assessments (VPAs) show promise as a means to measure complex non-cognitive constructs including resilience, and have been referred to as the next-generation of assessment (de-Juan-Ripoll et al., 2018; Hao and Mislevy, 2018; Lee et al., 2019). A recent scoping review found interactive technology can deliver effective interventions to increase resilience (Pusey et al., 2020). The primary aim of this paper is to detail the design, development and validation of an immersive simulation-based assessment methodology to measure resilience, focusing on different trajectories within an acute stressful event.

The assessment framework draws from principles of evidence-centered design (Mislevy et al., 2003; Mislevy, 2013) and embedded stealth assessment (Shute, 2011), and is informed by well-established theories of resilience (Carver, 1998; Richardson, 2002). We created a dynamic simulation environment, where players had to adapt to and overcome various unexpected and challenging events to complete the task objective. Performance was reflective of different trajectories, and these pathways were validated against an existing gold-standard self-report measure of resilience, the Connor-Davidson Resilience Scale (CD-RISC; Connor and Davidson, 2003). The present research makes several novel theoretical and empirical contributions. First, we advance research on how game design elements, coupled with an evidence-based assessment framework, can make powerful assessment tools. Second, we give evidence of patterns of trajectories in response to adversity, emphasizing a holistic approach to resilience. Third, we contribute to recent advances in Machine Learning as a technique to analyze complex datasets and predict individual differences. Practically speaking, our work addresses the potential for gamified assessments as a supplementary method for measurement.

### Assessment of Resilience

Given different conceptualizations of the resilience construct in the literature (as a *trait*, a *process*, and/or *outcome*; see Luthar et al., 2000; Fletcher and Sarkar, 2013; Fogarty and Perera, 2016 for reviews), varying methods have been used for its assessment. Common methods for the assessment of resilience include: (1) self-report scales and situational judgment tests (e.g., Windle et al., 2011; Pangallo et al., 2015; Teng et al., 2020), (2) indirect based on the presence of risk and positive adaptation (e.g., Luthar and Zelazo, 2003), and (3) measuring resistance or adaptation to negative life events, everyday stressors, or experimentally created stressors (e.g., Hildebrandt et al., 2016; Seery and Quinton, 2016). Despite the wide range of assessment formats, they are all evaluated against fundamental reliability and validity criteria. The psychometric properties of self-report methods of resilience are well-established, with some measures demonstrating excellent validity and reliability (e.g., CD-RISC; Connor and Davidson, 2003). Nevertheless, these scales are designed to capture resilience as a *trait*, although research has moved toward a broader *process* view, depicting the process through which internal and external factors interact to influence one’s response to adversity (Pangallo et al., 2015). These scales also tend to approach resilience in basic terms of presence or absence of psychopathology (Bonanno and Diminich, 2013). However, this approach neglects the *distribution* of individual differences in resilience, that is, the different types or variations in responses to adversity. Researchers have also highlighted the need to develop multimodal methods of resilience, including moving toward objectively verified assessments, rather than a sole focus on resilience as a personality-like variable (Bonanno, 2012; Pangallo et al., 2015). Some self-report measures of resilience also have problems with discriminant validity as they fail to separate from related constructs (Fogarty et al., 2016; Fullerton et al., 2021), prompting some authors to advocate for an integrative process model of resilience (Fullerton et al., 2021). Game-based and simulation assessments offer a potential supplementary method for assessing the process of resilience, capturing responses to adverse or stressful events to produce an outcome. Integrating such an approach with the use of traditional self-report scales such as the CD-RISC to capture stable individual differences, would provide a more holistic depiction of the resilience process.

Self-report measures can also be affected by faking such as malingering, self-deception and impression management, especially in high-stakes contexts (Barrick and Mount, 1996; Bensch et al., 2019). This can impact both construct and criterion-related validity. Response distortion can negatively affect the factor structure of measures through differential item functioning (Zickar and Robie, 1999), and lead to misinformed decisions and predictions (Rosse et al., 1998). Different methods have been developed to detect response distortion. A common method is to correct scores using social desirability or lie scales. Yet, researchers have questioned the construct validity of these scales as they may confound response style with trait measurement (Ellingson et al., 2007). Other methods in detecting intentional response distortion include forced-choice response options (Cao and Drasgow, 2019), eye-tracking (van Hooft and Born, 2012), response latencies (Holden et al., 1992), and sophisticated mathematical algorithms to adjust scoring (Lee et al., 2014). However, these methods do not remedy the problem of reducing multiple sources or opportunities to distort responses. More recently, psychophysiological markers measuring responses to external stimuli (e.g., skin conductance) have been employed to indicate or predict resilience (Winslow et al., 2015). For instance, Walker et al. (2019) found individuals who rated themselves higher on trait resilience habituated quicker to acoustic startle stimuli (i.e., showed a reduction in the amplitude in skin conductance on repeated presentations). Although such approaches show promise for supplementing traditional methods, these studies tend to be resource-intensive, resulting in relatively small sample sizes typically below 40 participants (Berkovsky et al., 2019).

### Using Games and Simulations to Assess Psychological Constructs

Modern technologies have allowed for the development of dynamic games and simulation environments (DiCerbo et al., 2017). These open-ended discovery spaces take many forms, including entertainment games, serious educational games, simulations, and virtual/augmented reality (see Marlow et al., 2016; Simmering et al., 2019; Taub et al., 2020 for reviews). Gamification is referred to as the use of game design elements in non-gaming contexts (Deterding et al., 2011). Games and simulations are defined as “a system in which players engage in an artificial conflict, defined by rules, that results in a quantifiable outcome” (Salen and Zimmerman, 2004, p. 80). In addition, Prensky (2001) includes goals, interaction, feedback, and representation as game design elements. In other words, a game necessarily involves creating an environment, objects, connections, rules and choices that allow the player to identify with the virtual character and immerse themselves into that game (Salen and Zimmerman, 2004).

#### Game-Based Assessment

The commercial use of game-based assessment has increased substantially in recent years. For example, personality (Barends et al., 2019; McCord et al., 2019) and non-cognitive constructs such as resilience, adaptability and flexibility (Georgiou et al., 2019; Nikolaou et al., 2019) have been assessed for personnel selection via games. However, the rapid expansion of commercial gamified assessments has attracted reservations about their psychometric properties. The lack of transparent and publicly available information about the design and data gathered from these instruments leave researchers with little evidence to evaluate their utility (Chamorro-Premuzic et al., 2016). Other criticisms include that they: lack a theory-driven design, tend to not be vetted in terms of scientific rigor, and have yet to demonstrate validity and reliability comparable with existing measures (Church and Silzer, 2016; Ihsan and Furnham, 2018).

Game-based assessments are also frequently used in educational settings, and their design is typically well-informed by evidence-based assessment frameworks. This type of assessment can support learning objectives and outcomes (Conrad et al., 2014; Hamari et al., 2014; Kapp et al., 2019). For instance, Shute et al. (2013, 2015) have demonstrated how games, coupled with evidence-based embedded assessment, can validly assess hard-to-measure constructs in educational contexts such as persistence (Ventura and Shute, 2013; DiCerbo, 2014), problem-solving, and creativity (Kim and Shute, 2015). Through log files, games allow us to record both the player’s final choices and the decisions made before arriving at that choice (i.e., product and process data). This gives a rich bank of data that typically cannot be captured by closed-ended assessment tools (Ifenthaler et al., 2012).

#### Simulation-Based Assessment

Simulations are defined as “any artificial or synthetic environment that is created to manage the experiences of an individual with reality” (Marlow et al., 2016, p. 415). They share similar characteristics to games, however, can be distinguished in that they attempt to represent real-life situations and environments (Narayanasamy et al., 2006; Sauve et al., 2007; Marlow et al., 2016; Lamb et al., 2018). The level of realism or fidelity of simulated worlds can range from low to high, depending on how well the system represents reality (Beaubien and Baker, 2004). For instance, artificial fantasy in games vs. hyper-realistic 3D audio-visual rendering and motion platforms in large scale simulations. Similarly, user response options can range from purely symbolic such as keystrokes and mouse clicks, through to more realistic controls such as joysticks, steering wheels and pedals producing physically plausible changes, and all the way to locomotion in free-roam virtual reality applications. Representational and physical fidelity are important design considerations, and the cost-fidelity trade-off is a well-known problem. Whilst high fidelity may appear to be desirable, meaningful play actually comes from the interaction between players and the system of the game (Salen and Zimmerman, 2004). Thus, both low-and high-fidelity systems can foster meaningful interaction.

Using a combination of simulated environments and embedded performance assessment techniques, early systems measured behavioral task performance with minimal interference attributable to measurement itself. For instance, The Strategic and Tactical Assessment Record (STAR; Graham et al., 1985), produced a comprehensive array of perceptual and information processing parameters comparable with common laboratory measurements. One of its key advantages was that “all measurement procedures were embedded within the operations required to play a computer game” (Graham et al., 1985, p. 643). Shute (2011) describe the concept of *stealth assessment*, which involves embedding assessment unobtrusively and directly into the fabric of the gaming or simulated environment. Another strength of simulation technologies is the ability to continuously gather complex behavioral or performance data at a fine grain size, dynamically and in real-time (Aidman and Shmelyov, 2002; Johnson et al., 2016). For instance, a player can be immersed in a 3D augmented reality experience and presented with complex objects that move and rotate in space. In addition to recording their responses, it is also possible to capture their body and gaze movements, and how they rotate the objects around them. This richness of data collection makes simulated scenarios very powerful assessment tools. In this regard, simulated environments can exceed the usual physical and cost-prohibitive boundaries of space and time.

Nevertheless, well-designed simulations have many challenges and require substantial effort in the design and iteration phases. Some of these challenges include: (1) crafting appropriate and accurate digital environments to elicit the constructs of interest; (2) making valid inferences about the individual’s behavior without disrupting the “free-flow” feel of the simulation; and (3) processing, synthesizing, extracting and interpreting the large quantities of data captured (de Klerk et al., 2015). The following sections detail how these challenges were addressed in designing the present simulation using an evidence-centered design framework.

### Applying Evidence-Centered Design in Simulation-Based Assessment

For a simulation to be valid, we must consider psychometric principles from assessment design frameworks. Evidence-centered design (ECD; Mislevy et al., 2003, 2015; Mislevy, 2013) provides an excellent point of departure. ECD builds an evidentiary chain of reasoning (DiCerbo, 2017) and dates to Messick (1994, 1995), who lays out a series of questions for assessment design: (1) what knowledge, skills, or attributes should be assessed (are they valued by society)? (2) what behaviors reveal those constructs? and (3) what tasks or scenarios should elicit those behaviors? The ECD framework builds on the vision of Messick by formalizing these questions through three central models in the Conceptual Assessment Framework: Student (or Competency) Model, Task Model, and Evidence Model (Mislevy et al., 2003). The Student Model answers the question of *What are we measuring?* It defines the variables related to the knowledge, skills and abilities to be measured. The Task Model answers the question *Where do we measure it?* That is, what tasks elicit behaviors to produce the evidence? It describes the environment or scenarios in which individuals say or do something to produce evidence about the target construct. Finally, the Evidence Model bridges the Student and Tasks Models and answers the question *How do we measure it?* That is, what behaviors reveal different levels of the targeted construct? It analyses a player’s interaction with, and responses to a given task. An evidence model consists of two parts: evidence rules and statistical model. Evidence rules take work product (e.g., a sequence of actions) that comes from the individual’s interaction with the task as input, and produce observable variables (e.g., scores) as output, which are summaries of the work product. The statistical model expresses the relationship between the constructs of interest in the Student Model and the observable variables.

#### The Student Model: Defining Resilience

The rationale for investigating resilience as the focal construct is because stressful and aversive events continue to plague humans at each stage of the lifecycle (Bonanno and Diminich, 2013). How one responds to setbacks and uncertainty is critical for well-being and survival in a constantly evolving world. Resilience entails two core concepts: (1) the presence of a potential stressor, and (2) positive adaptation (Fullerton et al., 2021). The term “potential” is important as there are differences in how individuals react to a certain event; some people are overwhelmed by daily hassles whereas others thrive in testing experiences (Bonanno, 2012). This means that people do not uniformly perceive a potential stressor as stressful. Recent research also emphasizes the broad term *stressor*, which encompasses a range of demands that necessitate resilience (Fletcher and Sarkar, 2013). Earlier definitions used the narrow term *adversity*, which only captures significant negative life events. Davis et al. (2009, p. 1638) argued that “for most of us, the adversities we encounter do not constitute major disasters but rather are more modest disruptions that are embedded in our everyday lives.” Thus, this study focuses on modest short-term stressors, rather than severe long-term hardship.

**FIGURE 1:**
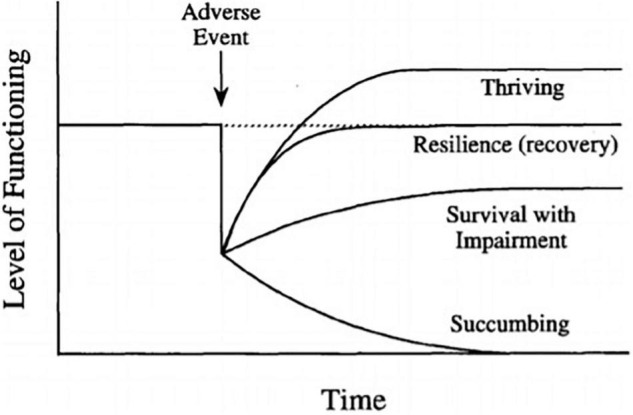
Responses to adversity. From “Resilience and thriving: Issues, models, and linkages” by Carver (1998), *Journal of Social Issues*, p. 246. Copyright 1998 by Wiley.

By integrating Carver’s (1998) responses to adversity framework and Richardson’s (2002) metatheory of resilience, four potential trajectories can result after experiencing a stressor: (1) resilient reintegration, where the individual returns to a higher level of homeostasis (thriving), (2) homeostatic reintegration, where the individual returns to their baseline level after decline (recovery), (3) reintegration with loss, leading to a lower level of functioning (surviving), and (4) dysfunctional reintegration, leading to maladaptive behaviors (succumbing). This is presented in Figure 1. In the present study, the model is applied to a discrete, time-bound adverse experience (although it can also be applied to a prolonged period of adversity; Carver, 1998; Bonanno and Diminich, 2013).

#### The Task Model: Design Elements

As mentioned earlier, the most common method to measure resilience is via self-report. A less commonly developed method is measuring resistance or adaptation to experimentally created stressors. Thus, we employed the latter method by aiming to capture responses to a clearly referenced adversity, where an individual is surrounded by high levels of emotional (and potentially physiological) stress. This “reactivity” approach involves measuring behavioral, subjective, or physiological responses to stimuli, whether naturalistic or experimental (Davydov et al., 2010). Adaptive reactivity would indicate resilience. The challenge was to foster high enough levels of stress and demand to elicit responses (not necessarily in magnitude, but in nature) to those experienced in real-world situations. To foster meaningful play, designing the formal system structures of the present simulation took into consideration core game elements, including objectives, procedures, rules, players and player interaction, conflict, and outcomes (Salen and Zimmerman, 2004).

The scenario was a driving simulation; however, it is important to note that the driving task is merely a medium or means to demonstrate the *method* of assessment (i.e., embedded evidence-based design framework), which is the focus of this research and described in detail below. That is, the assessment methodology is based on an ECD framework and intended to measure the target construct, thus should be independent of the medium. The driving scenario does not intend to measure driving or gaming experience *per se*, but rather, an individual’s trajectory in response to stressors, which would demonstrate pathways of resilience as hypothesized in previous theories of resilience. Indeed, any type of simulated task could be used (e.g., flight, racing simulators), so long as it follows and is grounded in a strong theoretical assessment framework and methodology. A driving task was chosen out of practicality because they are middle ground in representational and physical fidelity, with physically consequential responses captured via steering action and pedals for acceleration and braking.

**FIGURE 2:**
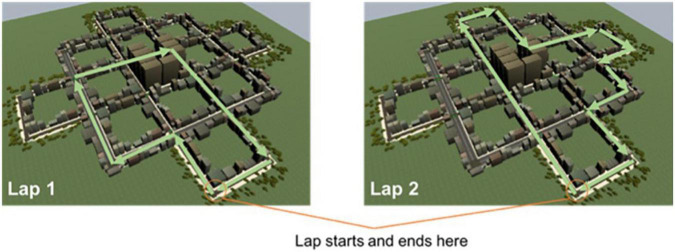
Examples of paths taken by the driver on two consecutive laps.

*Players* actively partook in a simulated training exercise for drivers of emergency vehicles (e.g., ambulance). Taking on the role of an emergency driver created a sense of urgency and time-pressure. Players drove around a metropolitan area designed as an urban grid, until they reached a final predetermined destination. This allowed players to be continuously directed along new routes in a relatively easily rendered space. Unbeknownst to them, they completed five different “laps” within the metropolitan area. Laps began and ended at the same location but took different paths around the city (see Figure 2).

**FIGURE 3:**
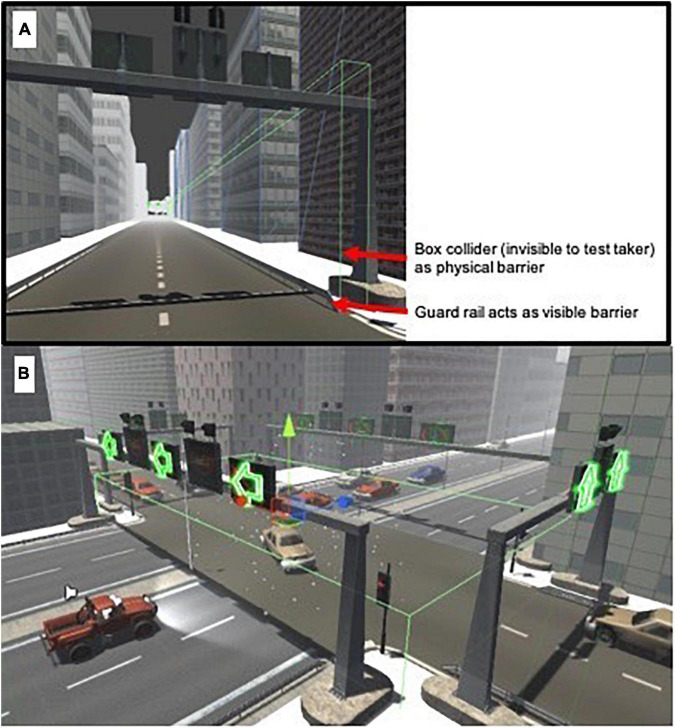
**(A)** Guard rails and box colliders used to keep players on the road. **(B)** Birds-eye view of a player (red vehicle) entering the unpreventable (black ice) event. Spatially, the event is bound by a trigger collider appearing as fluorescent green lines (invisible to players). Upon entering, tire friction is reduced to near zero, causing the vehicle to slide. Upon exiting, tire friction returns to normal.

*Procedures*, which are specific instructions of what actions to take, involved the player driving in a direction given by green arrows at every intersection. Directional arrows allowed the ability to create standardized and identical testing conditions. Hence, all players experienced the same events at approximately the same time. They could deviate from the arrows; however, they were instructed to stay on the desired path. Even if left unfollowed, participants could eventually return onto the directed path, although it would take longer for them to complete the task. If they did not follow the arrows, red no-go signs appeared, reminding them to make a U-turn which would lead back to the arrows. The *objective*, which aims to increase motivation and engagement, was to reach the destination as quickly and as safely as possible (i.e., maximize speed and minimize collisions). If present, another player controlled an Unmanned Aerial Vehicle (UAV) with a birds-eye view, in which they communicated information to the driver to help them drive faster and safer. For instance, instructing the driver when it was safe to use an incoming traffic lane. Depending on the helpfulness of the information, *player interaction* facilitated or inhibited the drivers’ actions. *Rules*, which define conditions, restrict actions and determine effects on players, specified what players could and could not do. Players could break road rules (e.g., speeding, driving in the opposite lane), and could only drive on the road (e.g., could not drive on footpaths or through parks). Small road guards were added to prevent cars leaving the road. Figure 3A shows how this was managed via large invisible colliders.

*Conflict*, which emerges to prevent players from achieving their goal, was another core element. The type of conflict were physical obstacles encountered throughout the drive—what we term as “event probes.” This event-based methodology is a systematic approach to designing simulated scenarios that are linked to the target constructs for assessment (Salas et al., 2009). Events must have clear start and end points to demarcate windows of meaningful data. Within these windows, the conditions players are exposed to are comparable. In line with stealth assessment, events were created and embedded with an emphasis on the scenario. Event probes appeared unexpectedly, disrupting players’ actions such that a decrease in speed and increase in collisions was likely. Five events were designed: a falling lamp post, animals crossing the road, a stationary car blocking the driving lane, dense fog, and black ice. For the first three events, drivers needed to slow down and/or change lanes to avoid collisions. For the dense fog, drivers needed to reduce speed as long-distance vision was compromised. For the black ice, there was a loss of friction causing the car to slide, meaning drivers had to persist in accelerating and maneuvering the position of the car to avoid collisions (see Figure 3B).

Each event was presented once in each lap, meaning participants had five encounters with each event throughout the entire simulation. The insertion of event probes changed task demands in which participants were to adapt and overcome. The onset of each event was without warning, and embedded at a different location and order on each lap, in order to mitigate associating an event with a certain location. This is known as the “task-change paradigm” (Lang and Bliese, 2009). Event probes also provoked stress and frustration, requiring players to maintain composure. Emotional regulation is needed to redirect situational attention toward task demands (Niessen and Jimmieson, 2016). When no event probes took place, this was referred to as “probe-free periods.” Nevertheless, participants still navigated heavy and changing traffic conditions. They also did not have an opportunity to practice as the aim was to capture dynamic responses under stressful and unpredictable conditions. In simulations aiming to capture other behaviors, it might be reasonable to inform players about the obstacles they are about to face and to provide them with the practice opportunities. This, however, would have compromised the purpose of the simulated environment.

#### The Evidence Model: Individual Performance Trajectories Using Slope Analysis

Log files record players’ progress throughout the game or simulation. Analyzing log files involves parsing for relevant information and extracting performance indicators (Greiff et al., 2016; Hao et al., 2016; Hao and Mislevy, 2018). Identifying evidence that connects performance to the target construct requires well-structured log files and analysis methods (Hao et al., 2016). Three types data were collected:

1. A tab-separated values file for session-level information (e.g., start time of the simulation, session ID, player ID);
2. A tab-separated values file for time-stamped actions (e.g., collisions with other cars);
3. A directory of tab-separated values files, one for each value time-stamped and recorded frame-by-frame (e.g., angle of the steering wheel; position of the vehicle).

Evidence of the target constructs need to reflect how participants respond to the event probes, and how their responses change over the course of the simulation with each encounter. Speed and collisions were included as the key output indicators. They were chosen as variables of interest as they give measurable, unambiguous and objective outcomes of performance level. A response-time in complex simulation-based tasks is generally suggested to be in scoring (Lee et al., 2019). Speed was defined as an arbitrary digital miles per hour, and collisions were measured as the number of times a participant’s vehicle collided with external objects. Below summarizes the process of how work product was taken as input (e.g., raw time-stamped data indicating a sequence of actions) and how observable variables (e.g., lap-level scores/measurements) were produced as output. This is known as the evidence rules.

**FIGURE 4:**
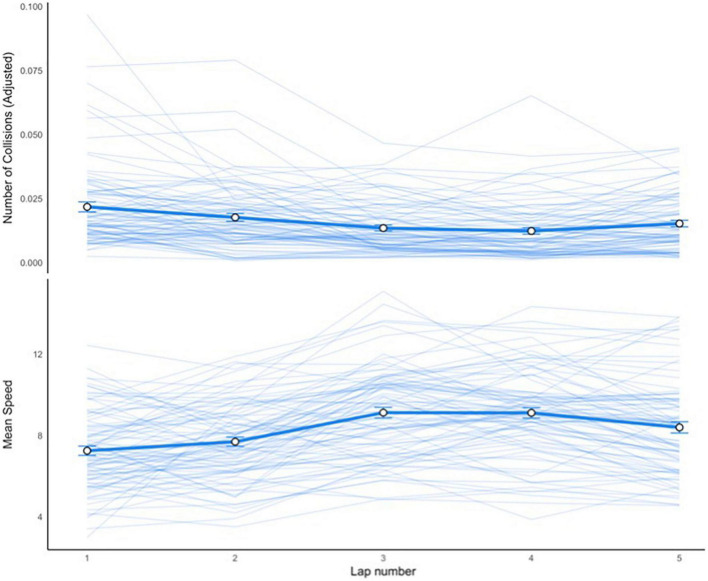
Mean speed per lap. The thick lines represent the mean for all drivers with error bars representing 95% confidence intervals. Each thin line represents a single driver’s number of collisions or average speed per lap. The number of collisions were adjusted to account for the different lap lengths. A ratio was calculated as the number of collisions per lap divided by the distance traveled per lap.

Step 1: Lap-level measurements. The raw timestamped data included logs of collisions, speed, frame-by-frame recordings of the vehicle position, and steering wheel angle, accelerator and brake values. These data were used to create the following lap-level scores: collision frequency, average speed, time taken, distance traveled, and the mean and standard deviation of steering wheel, accelerator, and brake values. That is, each driver had five estimates for each of these variables corresponding to each of the five laps. Average speed and collisions were also estimated overall (i.e., across the entire drive), during event probes, and during probe-free periods. Figure 4 shows the average speed and number of collisions of all drivers for each lap.

Step 2: Slope analysis. Derived variable analysis takes a collection of measurements (in this case, the five lap-level scores) and collapses them into a single meaningful summary feature (in this case, the slope) (Diggle et al., 2002). Slope analysis was used to determine individual performance trajectories. That is, we analyzed an individual’s rate of change over time, with the slope as the key outcome. For each individual, the magnitude and direction of performance changes (Y) were estimated over baseline (intercept), linear, quadratic, and cubic slopes (see Equation 1). The model intercept (α) and slopes (β_1_X_i_ *linear*, β_2_X^2^i *quadratic* and β_3_X^3^i *cubic*) were derived for each participant on each of the five estimates for both speed and collisions to represent a player’s starting point and change over time. The statistical significance of slopes (beta weights) capture the strength of changes. The performance trajectories are indicative of individual differences in responses to stress (defined in the Student Model): thriving, recovery, surviving, or succumbing. This is known as the statistical model.


(1) Y=α+β1X(linear)i+β2X(quadratic)2i+β3X(cubic)3i±ε

#### Slope Analysis: Linear, Quadratic, and Cubic Slopes

**FIGURE 5:**
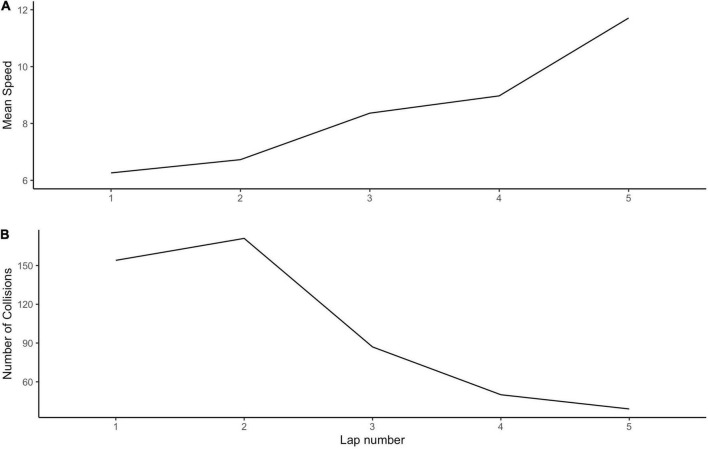
Examples of different slopes indicating thriving.

For speed, *thriving* is indicated by a strong positive linear slope (see Figure 5A). For collisions, *thriving* is indicated by a strong negative linear slope (see Figure 5B). These trends indicate the player continuously improved their performance (faster speeds and lower amount of collisions), despite obstacles encountered throughout the drive. These individuals adapted quickly to the changing situation; there was no or a relatively small loss of performance following obstacles or changes in the simulated environment in a lap 1, and they quickly relearned the changed situation. Thriving reflects decreased reactivity and faster recovery to subsequent stressful events, and a consistently high level of functioning (O’Leary and Ickovics, 1995; Carver, 1998). The strong the betas, the stronger the improvements across the five laps.

**FIGURE 6:**
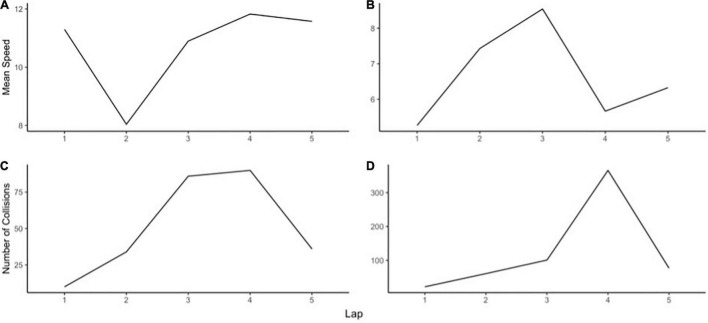
Examples of different slopes indicating recovery.

For speed, *recovery* is indicated by a strong positive quadratic beta, or a strong positive cubic beta (see Figures 6A,B). For collisions, *recovery*, is reflected by a strong negative quadratic beta, or a strong negative cubic beta (see Figures 6C,D). These trends demonstrate the ability to bounce back or return to former levels of functioning, after a decline in performance. After a downturn, they either return to baseline levels or continue toward an upward trend and function at a higher level than previously. After repeated experiences with the events, they are able to recover, should the stressor recur.

**FIGURE 7:**
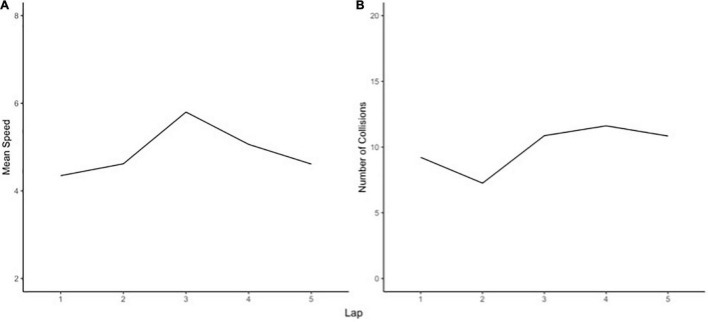
Examples of different slopes indicating surviving.

Weak or non-significant linear slope indicates merely *surviving* (i.e., strength of betas is relatively low). Whilst no significant improvements nor declines, these participants maintained homeostasis and were able to “just get past” the challenging events. Examples of surviving are displayed in Figures 7A,B for speed and collisions, respectively. These participants are able to withstand the challenging events, with no major deterioration nor improvement. The ability to withstand stressors is argued to be commonplace, emerging from the normative functions of human adaptation systems (Masten, 2001).

**FIGURE 8:**
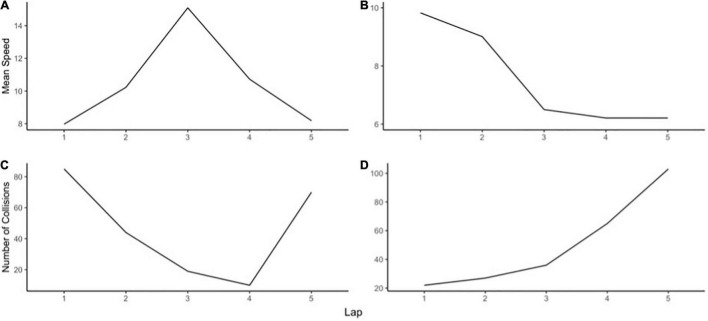
Examples of different slopes indicating succumbing.

Finally, *succumbing* is indicated by various slopes. For speed, strong negative linear, quadratic, or cubic slopes indicate a poorer performance level relative to the initial baseline level (see Figures 8A,B). For collisions, strong positive linear, quadratic, or cubic slopes indicate succumbing (see Figures 8C,D). These participants show a continued downward slide (slower speeds and greater amount of collisions). They have a relatively large loss of performance following unexpected events, and they slowly or are unable to adapt to them. This leads to eventual succumbing after experience with repeated stressful events.

#### Validation With an Existing Resilience Measure

Following the approaches of Ventura and Shute (2013) and DiCerbo (2014), the individual performance trajectories were validated against an existing measure of resilience, the CD-RISC (Connor and Davidson, 2003). Connor and Davidson (2003) adopted Richardson’s (2002) metatheory of resilience to develop their widely validated scale. It captures several trait-like aspects of resilience: the ability to adapt, to deal with stressors, to stay focus under stress, to handle unpleasant feelings, and the ability to stay on a task in the face of failure. The scale demonstrated strong psychometric properties. Reviews Windle et al. (2011) and Pangallo et al. (2015) have given the CD-RISC the highest rating in terms of quality criteria (validity and reliability), compared to other resilience scales. For validation, individual performance trajectories (linear, quadratic, and cubic slopes derived from lap-level measurements) are taken as inputs, and scores from the CD-RISC are taken as outcome criteria for predictive models. The experiment was conducted in a low-stakes environment to foster genuine responses on the CD-RISC, as the motivation to distort responses is more likely to occur in high-stakes situations such as job assessments (Donovan et al., 2014).

### Research Questions and Aims

The overarching goal of the present research was to design a simulation-based assessment methodology to measure psychological resilience and provide data to evaluate its merits. Given the same experiences with the challenging simulated scenario and events, which people thrive and which are impaired? This study focuses on two key research questions to assess the validity of the simulation-based assessment:

1. Can slope analysis (i.e., rate of change over time in the simulated task) give empirical evidence of resilience theories hypothesizing different individual trajectories of responses to stressors (thriving, recovery, surviving, succumbing)?
2. What is the relationship between individual trajectories and scores on an existing resilience measure (CD-RISC)?

## Methods

We report how we determined our sample size, all data exclusions, and all measures in the study.

### Participants

109 undergraduate students participated and acted as drivers in the simulation, in return for course credit (59 females, 50 males; mean age = 20.10, *SD* = 4.72). Two participants were excluded because they did not complete the simulation. Four participants were excluded because they ignored instructions to follow the arrows directing their course, thus missing multiple events (more than 7). An additional 13 participants were excluded due to a hardware error which caused their teammate to observe different traffic conditions to what they experienced, making the teammate’s instructions inaccurate. The final sample was composed of 90 participants who acted as drivers in the simulation (50 females, 40 males; mean age = 20.40, *SD* = 5.11). Machine Learning analyses (MLA) were utilized to quantify the accuracy of predictions. There are no agreed upon normative rules about how much data is needed to validate predictive models using MLA. Bzdok et al. (2018) recommends these analytics “when one is dealing with ‘wide data,’ where the number of input variables exceeds the number of subjects, in contrast to “long data” (2018, p. 233). The decision is typically based on the complexity of both the research question and the learning algorithm used in training and prediction, and the number of classes, input features, and model parameters used (see Raudys and Jain, 1991). Given the simplicity of the research questions and predictive model (based on eight main features used separately for two metrics—speed and collision), and standard algorithms used, this sample size is more than satisfactory for the preliminary validation of the simulation before proceeding with a cross-validation of these results. This sample size was also adequate to examine a baseline linear regression model (with more than 10 people per feature/variable). More details are provided in the Machine Learning Analysis section of the results below.

### Measures

#### Simulation and Related Measures

##### Driving Simulation

The simulation is described above in the introduction.

*NASA Task Load Index (NASA-TLX;* Hart and Staveland, 1988). This is a 6-item measure of workload. It was administered immediately after, and in direct relation to the simulation. An example item is, “How mentally demanding was the task?” which was rated on a 7-point scale from (1) *very low* to (7) *very high*.

##### Simulator Sickness (Kennedy et al., 1993)

This questionnaire measures 16 symptoms of simulator sickness and cybersickness. There were three symptom clusters including Oculomotor (e.g., eyestrain, headache), Disorientation (e.g., dizziness, difficulty concentrating), and Nausea (e.g., stomach awareness, burping). Symptoms were scored on a 4-point scale from (0) *None* to (3) *Severe*. This questionnaire has demonstrated good internal consistency estimates (0.87; Bouchard et al., 2007).

##### Driving Experience and Gaming Intensity

Driving experience was recorded as the number of years driving a car, and gaming intensity was measured as the average number of hours spent playing video games in a single session.

#### Validation Measure

*Connor-Davidson Resilience Scale (CD-RISC;* Connor and Davidson, 2003). This measure consists of 25 items assessing the ability to cope with stress and adversity. Items (e.g., “I am able to adapt to change”) were rated on a 5-point Likert scale from (1) *not true at all* to (5) *nearly always true*. A higher total score indicates greater resilience. This scale has demonstrated good internal reliability (0.89; Connor and Davidson, 2003).

### Procedure

The driving simulation was hosted on Unity game engine platform,^1^ and presented on LG flat-screen monitors (43-inch screen size). The driving station consisted of Logitech G920 Driving Force Racing Wheel, Pedals and Playseat. All other measures were computerized and hosted on Qualtrics, a survey software platform. Sixty-seven (74.4%) drivers did the simulation with a teammate and twenty-three (25.6%) drivers completed it alone. Participants did not know each other before the study. They first completed demographic information (age, gender) and the simulation-related measures. They then completed the driving simulation (30 min), followed by the CD-RISC. Ethics approval was granted by Australian Defence Science and Technology Group Low-Risk Human Research Ethics Review (Protocol Number 07/415).

## Results

We begin by presenting descriptive statistics, providing a comprehensive examination of simulation-derived metrics, self-reported resilience, and evaluation of the simulation. Next, we present results of the slope analyses, including the proportion of individuals showing different performance trajectories. Given the complex distributional properties of the simulation-derived metrics and possible non-linear relationships between variables, we then used Machine Learning Analysis (MLA) to quantify the accuracy of predictions (Berkovsky et al., 2019; Jacobucci and Grimm, 2020). MLA was employed instead of traditional correlational analyses, because it can achieve relatively greater sensitivity compared to conventional techniques, which would likely deflate and/or fail to capture relationships between the variables (Koul et al., 2018). As MLA in psychological sciences is concerned with predictive accuracy, to optimize prediction we compared algorithms across degrees of complexity through the use of different linear and non-linear algorithms (including ridge regression, support vector machines, boosting, random forests) to examine relationships in the data (see Yarkoni and Westfall, 2017; Dwyer et al., 2018; Koul et al., 2018; Bleidorn and Hopwood, 2019; Jacobucci and Grimm, 2020; Orrù et al., 2020 for reviews). Accuracy of these algorithms were also compared to a baseline linear regression model to test whether they outperform the baseline.

### Descriptive Statistics

#### Simulation-Derived Metrics

**FIGURE 9:**
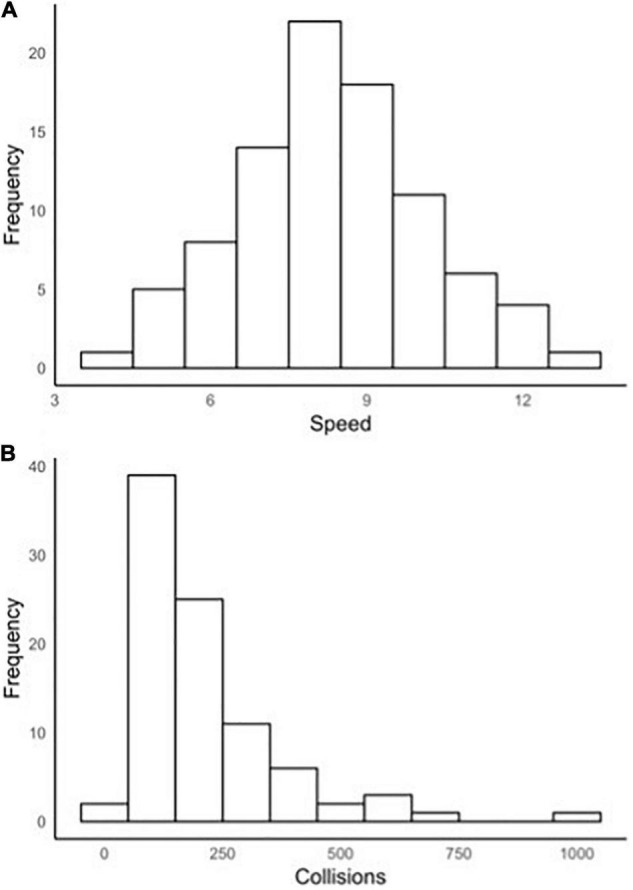
**(A,B)** Histogram for A. speed & B. collisions.

Figures 9A,B display the frequency distributions for overall speed and collisions in the simulation. Speed was normally distributed whilst collisions were positively skewed.

Table 1 presents descriptive statistics and Cronbach’s alphas for average speed and number of collisions overall, during probe-free periods and during event probes. Internal consistency for speed and number of collisions overall across the five laps was high, and acceptable to good during probe-free periods and event probes. Paired sample *t*-tests were conducted to examine differences in speed and collisions during event probes vs. during probe-free periods. Participants had more collisions during event probes (*t* = 4.05, *p* < 0.001, Cohen’s *d* = 0.43), however, there were no significant differences in average speed (*t* = –1.42, *p* = 0.16, Cohen’s *d* = –0.15).

**TABLE 1:** Descriptive statistics for simulation-derived metrics.

	Mean	*SD*	Range	Alpha	*t*-value	Cohen’s *d*
**Speed**						
Overall	8.32	1.79	4.36–12.76	0.91		
Probe-free periods	9.29	2.12	4.98–14.93	0.87	–1.42	–0.15
Event probes	9.05	2.09	4.99–15.21	0.79		
**Collisions**						
Overall	211.77	162.49	31–966	0.80		
Probe-free periods	94.41	92.24	9–464	0.76	4.05***	0.43
Event probes	117.36	78.34	22–502	0.69		

***p < 0.001. Speed is reported as the average digital units per hour across the five laps. Collisions is reported as the total number of collisions across the five laps. For subsequent analyses using collisions, a ratio was calculated as the number of collisions per lap divided by the distance traveled per lap, to account for different lap lengths.

### Simulation-Related Measures

Table 2 presents the descriptive statistics for NASA-TLX, driving experience, and gaming intensity. It appeared that participants were highly engaged and motivated to try their best in following the instructions to achieve the goal. Participants reported the highest score for effort, followed by mental and temporal (time-pressured) demands, then frustration, physical demand, and finally for performance.

**TABLE 2:** Descriptive statistics for NASA-TLX, driving experience and gaming intensity.

Measure	Mean	*SD*	Range
**NASA-TLX**			
Effort	5.23	1.15	2–7
Frustration	3.83	1.78	1–7
Mental demand	4.84	1.39	1–7
Performance	3.20	1.10	1–5
Physical demand	3.80	1.60	1–7
Temporal demand	4.93	1.41	1–7
**Driving and gaming**			
Driving years	3.45	4.82	0–30
Gaming hours	1.24	1.09	0–5

Table 3 summarizes paired sample *t*-tests comparing pre- and post-simulation for 16 simulator sickness symptoms. Participants reported changes to half of the symptoms. Cohen’s *d* were below medium effect sizes (*d* < 0.50) for 75% of affected symptoms. The most potent differences were sweating and fullness of the head with *d-*values over a medium effect size. The *Nausea* cluster of symptoms was most affected (in order of effect sizes): sweating, general discomfort, salivation, and nausea. The second most affected cluster was Disorientation (in order of effect sizes): fullness of head, and dizziness (eyes open). Two symptoms in the Oculomotor cluster (eyestrain, headache) were affected but their effect sizes were small. Participants reported no differences in fatigue, difficulty focusing and concentrating, blurred vision, dizziness (eyes closed) vertigo, stomach awareness, or burping.

**TABLE 3:** Paired sample *t*-tests evaluating differences between simulator sickness symptoms pre- and post-simulation (*N* = 90, df_89_).

	Mean pre	SD pre	Mean post	SD post	Mean diff.	SE mean diff.	*t*-value	Cohen’s *d*
General discomfort	1.11	0.38	1.33	0.60	-0.22	0.07	–3.01**	0.40
Fatigue	1.24	0.53	1.34	0.62	–0.10	0.08	–1.32	–
Headache	1.07	0.25	1.18	0.41	–0.11	0.04	–2.58*	0.28
Eyestrain	1.16	0.42	1.29	0.57	–0.13	0.06	–2.16*	0.31
Difficulty focusing	1.11	0.35	1.17	0.46	–0.06	0.05	–1.15	–
Increased salivation	1.03	0.18	1.19	0.52	–0.16	0.05	–2.85**	0.38
Sweating	1.22	0.51	1.64	0.89	–0.42	0.09	–4.52**	0.60
Nausea	1.04	0.21	1.20	0.56	–0.16	0.06	–2.64*	0.35
Difficulty concentrating	1.10	0.40	1.17	0.43	–0.07	0.05	–1.23	–
Fullness of head	1.06	0.27	1.29	0.62	–0.23	0.07	–3.58**	0.53
Blurred vision	1.04	0.21	1.12	0.39	–0.08	0.04	–1.83	–
Dizziness (eyes open)	1.03	0.18	1.13	0.34	–0.10	0.04	–2.57*	0.36
Dizziness (eyes closed)	1.11	0.35	1.23	0.56	–0.12	0.07	–1.83	–
Vertigo	1.04	0.21	1.10	0.40	–0.06	0.04	–1.39	–
Stomach awareness	1.11	0.35	1.20	0.48	–0.09	0.05	–1.72	–
Burping	1.03	0.23	1.06	0.23	–0.02	0.03	–0.82	–

*p < 0.05; **p < 0.01.

An overall simulator sickness score was computed both pre- and post-simulation. The difference between the two scores was standardized. Majority of drivers (83.3%) were within 1 SD of the mean difference symptoms score. Four drivers (4.4%) reported an improvement in symptomatology in the size of 1 (3 drivers) and 3 (1 driver) SDs, respectively. Four drivers (4.4%) were 1 SD below the mean of the standardized difference score. Five and two drivers were 2 and 3 SDs below the mean, respectively. Thus, these seven people reported a notable detrimental change in symptomatology post-simulation.

### Validation Measure

**FIGURE 10:**
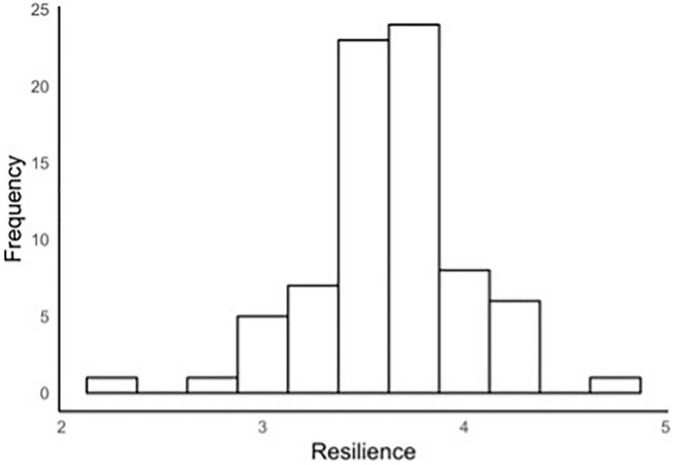
Histogram of CD-RISC scores.

The mean score on the CD-RISC was 3.63 (*SD* = 0.40, Range = 2.20–4.84) and internal consistency was good (α = 0.86). The frequency distributions represented a good spread of variance, rather than a skewed distribution (see Figure 10). In low-stakes situations such as that of the present research, people are less inclined to “fake good” or “fake bad,” thus a normal distribution was observed, with an expected proportion of people reporting relatively low or high resilience levels.

### Slope Analysis: Individual Performance Trajectories

Table 4 summarizes the percentage of people with different slopes for speed and collisions, during both the event probes and probe-free periods. Significant positive and negative slopes were investigated for the linear, quadratic, and cubic slopes. To allow for marginal error, the significance level was set at *p* = 0.20. Several findings are worth noting.

**TABLE 4:** Percent (%) of participants with significant slopes (*N* = 90).

		Speed		Collisions	
Slope		Probe-free periods	Event probes	Probe-free periods	Event probes
Linear	Positive	14.44	11.11	11.11	7.78
	Negative	16.67	4.44	8.89	15.56
Quadratic	Positive	10.00	5.56	12.22	12.22
	Negative	11.11	12.22	6.67	8.89
Cubic	Positive	11.11	10.00	11.11	6.67
	Negative	11.11	5.56	2.22	8.89

During the event probes, a small proportion of people had a positive linear slope for speed and a negative linear slope for collisions, which is indicative of *thriving*. With each encounter with an event, performance improved (speed increased, collisions decreased). These participants displayed a consistently high level of functioning despite constant challenges. During the event probes, a small proportion of people displayed positive quadratic and cubic slopes, respectively, for speed, and negative quadratic and cubic slopes, respectively, for collisions. These trends suggest the ability to *recover*—these people experienced a brief downward slide in performance but were able to bounce back from the embedded stressors and returned to their previous levels of functioning. A large proportion of participants (around 60–70%, with the percent varying for speed and collisions during events and probe-free periods) displayed non-significant trends (i.e., relatively weak betas or plateau-like slope). This suggests they were merely *surviving*—their performance level neither substantially increased nor decreased, but they maintained homeostasis. Finally, during the events, a minor proportion of people showed a negative linear slope for speed and a positive linear slope for collisions. This may suggest *succumbing*—these participants failed to adapt their behavior to each recurring event and could not recover after initial poor performance. With each encounter with events, there was a downslide in performance.

### Machine Learning Analysis

One of the major applications of Machine Learning in psychological research is the development of models focused on predicting human behavior (Gonzalez, 2020). These analytics are recommended when *non-linear* relationships with well measured predictors are being modeled, which is a case for this research (Jacobucci and Grimm, 2020). Key machine learning techniques were considered in this study, including feature selection, cross-validation, and models/algorithms used. Feature selection is selecting specific variables from a larger dataset to enhance accuracy and generalizability (Dwyer et al., 2018). Eight speed features and eight collisions features were selected to train models to predict scores on the CD-RISC for validation. The eight features were: first lap value, best lap value, worst lap value, overall average lap value, intercept term, linear term, quadratic term, and cubic term. For each of these features, there were estimates for during the event probes, during probe-free periods, and throughout the simulation overall. In order to mitigate the risk of overfitting, we used a train-test split to derive train and test subsets (7:3 ratio of train:test). The hyperparameters were only trained in the train subset, and the results were for the test subset only.

A baseline regression model was first conducted using a linear regression; the outcome variable was separately regressed onto the eight features. A baseline model provides a reference point to which to compare different machine learning algorithms, including the extent to which these algorithms add improvement over and above the baseline (Brownlee, 2014). Then, three common linear and non-linear Machine Learning algorithms were applied: Random Forest, Bayesian Ridge regression and Support Vector Machine. Jacobucci and Grimm (2020) have recommended to compare predictive accuracy across algorithms with different degrees of complexity. The models were fitted and tested with predictions, and the Symmetric Mean Absolute Percentage Error (SMAPE) was used to quantify prediction accuracy. SMAPE is a widely used measure of accuracy, due to advantages of interpretability and scale-independency (Kim and Kim, 2016). These estimates are presented in Table 5. Each algorithm performed substantially better than the baseline linear regression model. While all methods produced good SMAPE estimates, attesting to the stability of the predictions, Bayesian Ridge regression algorithm produced the highest predictive accuracy (lowest error, with SMAPEs below 5%).

**TABLE 5:** Machine learning analysis: Predicting CD-RISC scores.

	Speed				Collisions			
	Probe-free periods		Event probes		Probe-free periods		Event probes	
Model	SMAPE	*SD*	SMAPE	*SD*	SMAPE	*SD*	SMAPE	*SD*
Baseline	7.90	7.63	7.91	7.08	7.78	7.11	7.92	7.12
Random forest	4.52	3.89	4.92	4.18	5.50	4.36	5.02	4.82
**Bayesian Ridge**	**4.04**	**3.91**	**3.80**	**2.80**	**3.85**	**5.15**	**3.21**	**2.74**
Support vector machine	4.22	4.16	4.42	5.32	4.13	5.68	3.63	3.60
**Bayesian Ridge for**								
Driving years	46.04	32.52	36.84	27.86	40.46	30.83	40.34	29.32
Gaming time	37.47	23.57	41.46	32.68	36.57	30.65	50.15	35.97

*SMAPE, symmetric mean absolute percentage error. The best performing algorithm is bolded.*

To demonstrate that the methodology was an assessment of resilience, rather than driving or gaming ability, both driving experience and gaming intensity were investigated as outcome variables in the machine learning models. The bottom of Table 5 also presents the SMAPE estimates using the best performing algorithm, Bayesian Ridge regression (see Supplementary Material for results of other algorithms and baseline). Individual performance trajectories were weakly associated with driving experience, as it was predicted with relatively high error rates between 36.84 and 46.04%. Similarly, weak association was observed with gaming intensity: predictive error rates were high (between 36.57 and 50.15%). This suggests the simulation was assessing the target construct, resilience, and provides evidence of discriminant validity.

## Discussion

As modern technologies continue to progress, game and simulation design has correspondingly expanded for learning, assessment and training purposes. The present study aimed to detail the development, design and initial validation of a simulation-based assessment methodology to measure psychological resilience. The development of the simulation was guided by well-known resilience theories (Carver, 1998; Richardson, 2002), in addition to an evidence-centered design framework (Mislevy et al., 2003) and embedded stealth assessment (Shute, 2011). This study took a “reactivity” approach to measuring resilience, which involved systematic and deliberate exposure to stressful conditions (Davydov et al., 2010). These tests of reactivity to acute stressors have been proposed to assess different levels of adaptive or maladaptive responding. The findings demonstrate how game design elements, such as objectives, procedures, rules, and conflict, can be applied to make powerful assessment tools (Salas et al., 2009). Majority of players accomplished the objective of following arrows to reach the end destination. The success of this objective varied, with prominent individual differences in both absolute performance (speed and collisions) and trajectories (rate of change over the five laps). Conflict, crafted from the event probes, increased task difficulty by impeding players in reaching the objective of maximizing speed and minimizing collisions. Indeed, players collided more often during event probes, compared to probe-free periods. Players also reported the task being effortful, mentally demanding, and temporally demanding (fast-paced).

Performance in the simulation was recorded into log files unobtrusively—a key component of stealth assessment. Evidence extracted from these log files was used to identify different individual performance trajectories. That is, using derived variable analysis (Diggle et al., 2002), data from log files were transformed into lap-level measurements (i.e., average speed and collisions for each of the five laps), which were then collapsed into a meaningful summary feature—the slope. An individual’s slope indicated their rate of change throughout the simulated task. Using slope analysis, different trends were observed, including linear, quadratic, and cubic slopes in both positive and negative directions. The strength of the slopes (i.e., betas) also held important information. One research question we aimed to investigate was whether these slopes indicated different responses to stressors, which represents a holistic approach to the resilience process (Carver, 1998; Richardson, 2002). We used MLA to build predictive models of resilience based on the simulation-embedded metrics. These analytics are especially recommended when non-linear relationships with well measured predictors are being modeled (Jacobucci and Grimm, 2020). Both are the case in this research.

For the simulation-embedded metrics, this indicated the stability of speed and collision tendencies of drivers within the simulation. Secondly, it appeared that a small proportion of participants demonstrated *thriving*, indicated by a positive linear slope for speed and a negative linear slope for collisions. Under stressful conditions, they were able to maintain emotional regulation and composure, and had no or minimal performance decrements. Another small percentage of people displayed *recovery*, shown by positive quadratic and cubic slope for speed and negative quadratic and cubic slopes for collisions. These people recovered from an initial setback in performance and returned to their previous level of functioning. The majority of people showed *survival*, as they had no significant slope (e.g., plateau-like slope). They were able to withstand the stressors and showed adaptive behavior to maintain their performance level. This process of survival and minimal impact has been argued to be commonplace and arises from the basic, normative functions of human adaptation systems (Masten, 2001; Bonanno and Diminich, 2013). Hence, it is not surprising that a large proportion of people showed the ability to maintain homeostasis. Finally, a small proportion of people showed trends indicative of *succumbing*. They were unable to adapt to the changing conditions or bounce back after initial poor performance, possibly indicating maladaptive reactivity.

The findings have practical implications for simulation-based training to support resilience, particularly for those showing a trajectory of succumbing, or those who seek to improve their resilience in the face of adversity (see Pusey et al., 2020, for a review). Existing resilience training programs have shown promise in contexts such as defense, workplace, and medical (see Leppin et al., 2014; Joyce et al., 2018 for reviews). There is potential to employ the present simulation-based assessment methodology in conjunction with resiliency programs—a randomized controlled design with follow-up measurements can ascertain the effectiveness of such training programs on raising performance levels to a point of thriving. Few studies have investigated this approach of building adversity into resilience training programs by systematic exposure to realistic simulations. To illustrate, in studies by Arnetz et al. (2009) and McCraty and Atkinson (2012), first-responders participated in realistic simulation scenarios (e.g., high-speed car pursuits). Compared to a control condition, those in resilience training programs reported less psychophysiological stress and better performance in the simulation. Indeed, moderate exposure to adversity with appropriate challenges may help individuals develop resilience, particularly for future stressful situations (Robertson et al., 2015). Thus, combining simulation-based assessment and training may be a promising paradigm to building resilience.

Another research question we aimed to answer was how these individual slopes would relate to an existing self-report measure of resilience (CD-RISC), for purposes of validation. Using machine learning techniques, the individual response trajectories were predictive of CD-RISC scores with high accuracy, provides evidence of construct validity. Error rates were below 5% for the best performing algorithm, Bayesian Ridge regression; and, importantly, were similar for other algorithms used, attesting to the stability of the predictions. Each of the machine learning models used outperformed a baseline linear regression model also tested in terms of predictive validity. Hence, as recommended by Jacobucci and Grimm (2020), machine learning approaches are more sensitive to modeling non-linear relationships, which can complement traditional statistical analysis techniques. To demonstrate discriminant validity, driving experience and gaming intensity were also placed in the models as outcome variables. These variables were predicted with relatively high error rates (above 35%), implying that behaviors in the simulation was not necessarily sensitive to the reports of driving or gaming ability. Thus, we reiterate that it is the design and validation of the *assessment methodology*, and *not* the driving task, which was the focus of this research. No predictions about the actual driving skills and abilities can be made based on the simulation. The driving scenario was merely a convenient medium to demonstrate how ECD could be applied to assess different resilience trajectories in response to stressors. It is of course possible and needed for future research to develop and test other simulated tasks using a variety of mediums (e.g., flight simulators), embedding the methodology presented to demonstrate that the method of assessment is independent of the medium.

These findings advance the way we construe resilience by demonstrating the dynamic process through which individuals adapt to stressors. The prediction of CD-RISC scores of trait resilience from the trajectories supports the role of stable individual differences in shaping one’s adaptation to adverse events. However, the simulation goes beyond capturing these stable traits to assess real-time responses to stressors, i.e., the in-lab window into a *process* of resilience. We propose that capturing both is necessary to deepen our understanding of the psychological resilience construct.

### Challenges, Limitations, and Future Directions

Despite holding promise as an alternative or supplementary assessment method, gamified assessments are still in its early stages. The present study is a step in the direction toward a “next-generation” of assessment. However, there is a need for further validation with other well-validated measures of resilience, as well as with measures of similar constructs in the nomological network (e.g., adaptability), and related real-world outcomes (Aidman, 2020). This newly developed simulation methodology must also be tested and replicated across multiple samples and contexts to determine the generalizability of the findings.

A significant challenge encountered in this study was the need for multidisciplinary expertise. For example, development of the simulation required software developers, game developers and 3D modelers; data management and analysis (particularly predictive modeling) required data scientists and programmers; and understanding and implementing the theory and constructs required psychologists and cognitive scientists. Not only must these personnel have expertise in their respective areas, but they must also develop their work output with consideration for other experts. For example, the software developer must program the simulation to output data logs which capture the target constructs as defined by the research psychologist. These data logs must also be suitably formatted for use by the data scientist.

Another challenge relates to issues of accessibility and feasibility, including the need for specialized equipment (both software and hardware). The present simulation used Unity development platform, however there are many other game engines such as Bohemia Interactive Simulations, Unreal Engine, and Godot. Specialized hardware (e.g., driving equipment, virtual reality headsets and equipment) can also increase costs and the need for dedicated space. Since game- and simulation-based assessments are more expensive and can take greater time to develop and validate, researchers must weigh the costs and benefits about their needs and goals. Thus, it remains a future research direction to explore ways to create gamified assessment protocols that are accessible, accurate, and cost-effective for both researchers and end-users, so their full benefit can be realized.

Specific to driving and other motion-based simulations, the potential impact of simulation sickness on performance must be fully investigated to limit its severity. High levels of simulator sickness can affect performance by confounding data and influence participant dropout rates (Brooks et al., 2010). Indicated by the simulator sickness questionnaire measured pre- and post-simulation, majority of the sample (83.3%) were not affected by the simulation. However, 7.8% of the sample reported a notable increase in symptoms. About 50% of the 16 symptoms showed significant changes, but only 25% of them were of notable effect size. Moreover, while five laps were sufficient to examine rate of change over time, the stability of the slope analysis could be strengthened by increasing the number of laps. However, longer exposures can produce more symptoms (Kennedy et al., 2000; Brooks et al., 2010). Perhaps in future iterations, the lap length could be reduced and consequently, the number of laps could be increased, without increasing the duration of the task. Additional research is also needed to determine the optimal length of a single exposure.

While we focused on speed and collision metrics, other indicators could be used to measure resilient responses. This could include, for instance, intentional lane-changing strategies (data points that collect the location of the vehicle), and maintaining emotional composure (i.e., modulating the level of control over one’s responses to match environmental demands). Recording psychophysiological (e.g., skin conductance, heart rate variability) whilst completing the simulation may also give valuable information. Several studies have investigated indicators of physiological arousal whilst participants completed a stressful laboratory task (see Walker et al., 2017, for a review). For instance, Hildebrandt et al. (2016) placed participants in a threatening and changing immersive virtual environment while recording skin conductance, and found self-rated resilience predicted arousal during the sustained experience of threat. Other studies have investigated regulation and recovery from stressors via startle responses (Walker et al., 2019) or matching emotional responses to changing stimuli (Waugh et al., 2011). Employing biomarkers in conjunction with game- and simulation-based assessment protocols can act as an additional source of validation. It would be interesting to determine whether those with performance levels indicating thriving or recovery show better regulation of psychophysiological arousal (e.g., lower skin conductance)—this may provide evidence that resilient people can regulate and change their affective and physiological responses to match the demands of changing environmental circumstances.

This study recruited university students in a low-stakes context. While the sample size was appropriate, a larger sample is recommended to replicate these results. The promising aspect of this research is the stability of predictions across different ML algorithms; thus we anticipate that these results will replicate on a larger sample. Also, whilst there are limits of generalizability due to the sample characteristics, the results still show promising utility of the simulation-based assessment. Future studies should examine specific samples where resilience is critical for success (e.g., elite athletes, defense personnel, business managers). On a related note, the incremental and criterion-related validity of this methodology is yet to be established, above and beyond existing measures of resilience. Iterative validation of game- and simulation-based assessments includes determining their utility in predicting real-world outcomes (and being implemented in high-stakes environments; Georgiou et al., 2019; Nikolaou et al., 2019). These outcomes could be subjective or objective, for instance, attrition rates and posttraumatic stress trajectories in military personnel (Bonanno, 2012); game performance consistency and injury rehabilitation in competitive athletes (Sarkar and Fletcher, 2013); or job performance and burnout in employees such as healthcare professionals (Robertson et al., 2015). Finally, Machine Learning approaches typically require large sample sizes to train the data. Future research is recommended to obtain larger samples to strengthen model predictions.

## Conclusion

This work is contributing to the growing literature on gamified psychometrics, and to the theory of mental resilience, integrating the process model of resilience to its measurement. Game- and simulation-based assessment is a nascent research area, with promising progress being made toward their theory, design, validation, and implementation for end-users in various contexts (e.g., education, defense, organizational). Well-designed games and simulations provide opportunities to assess “hard-to-measure” constructs, particularly those regarded as twenty-first century skills (OECD, 2018); not to replace, but to supplement traditional measures and methods. Data can be collected continuously and unobtrusively (stealth assessment), providing a rich bank of information about individuals’ skills, abilities, and attributes. However, iterative, and rigorous validation is necessary for the utility of gamified assessments to be fully achieved. We look forward to the continued investigation of gamified methods that may change how we think about assessment.

## Data Availability Statement

The datasets presented in this article are not readily available because the release will need to be approved via DSTG internal review process. Requests to access the datasets should be directed to corresponding author.

## Ethics Statement

The studies involving human participants were reviewed and approved by the Defence Science and Technology Low-Risk Human Research Ethics Panel (Protocol Number 07/415). The patients/participants provided their written informed consent to participate in this study.

## Author Contributions

SK, SJ, and EA designed the study. SJ built the simulation. SJ, MB, and LZ collected the data. SK, SJ, MB, and NR analyzed the data. SJ, LZ, and SK wrote the manuscript. SK and EA revised the manuscript. All authors contributed to the article and approved the submitted version.

## Conflict of Interest

The authors declare that the research was conducted in the absence of any commercial or financial relationships that could be construed as a potential conflict of interest.

## Publisher’s Note

All claims expressed in this article are solely those of the authors and do not necessarily represent those of their affiliated organizations, or those of the publisher, the editors and the reviewers. Any product that may be evaluated in this article, or claim that may be made by its manufacturer, is not guaranteed or endorsed by the publisher.

## Footnotes

1 The simulation was originally designed to be Virtual Reality (VR) ready, and was piloted using the commercially released Oculus Rift on members of our research lab. However, we withdrew VR from testing of the main sample, due to virtual reality motion sickness. Whilst no participant in the pilot was physically ill, other symptoms (headaches, dizziness) from VR were adverse enough to interfere with the study.
