# Supplementary material for: Applying Evidence-Centered Design to Measure Psychological Resilience: The Development and Preliminary Validation of a Novel Simulation-Based Assessment Methodology
Source: Front Psychol. 2022 Jan 10;12:717568. doi: 10.3389/fpsyg.2021.717568 (PMC8786081; doi:10.3389/fpsyg.2021.717568)
Supplement: Supplementary file 1 [file Table_1.DOCX]

**Supplemental Material**

**Supplemental A: Verbal instructions given to simulation test-takers**

*<< Participants seated away from computers* >>

You are about to take part in a simulated training exercise for drivers of emergency vehicles like an ambulance or police car.

[*If drone operator available*]

One of you is the driver, and the other is a drone operator.

[*To driver*]

As the driver, your goal is to drive around the city in the direction given by the green arrows as quickly, but as safely, as possible. In other words, try to drive as fast as you can without hitting anything. Like an emergency vehicle, you’re allowed to ignore road rules. This means you do things like drive on the wrong side of the road or go through red lights. Remember, you have to follow the green arrows. If you ever see red no-go signs, this means that you’ve missed a turn. If this happens, do a U-turn and drive back until you see the green arrows again.

[*To drone operator*]

As the drone operator, you will control a camera mounted to a drone flying above the driver. This means that you’ll have a birds-eye view, and can provide the driver with information that can help them to drive faster and safer. In general, try to communicate information about traffic. For example, where are the cars ahead of the driver? Are there any gaps that the driver can take advantage of, and so on?

[*If drone operator*] [*To the driver*]

As the driver, you can ask the drone operator to provide you with whatever information you feel will help you most. So, if you want more, less, or different information than the drone operator is already giving you, be sure to communicate this to them.

<< *Seat participants at the computers and give them headsets* >>

[*If drone operator*]

If you can hear me, please give a thumbs up.

You will be able to communicate with me and each other. Driver, to talk to the drone operator, you must press and hold the ‘A’ button on your steering wheel. Drone operator, to talk to the driver, you must pull and hold the trigger on your joystick.

I will now begin setting up the simulations on your computers. Please check that you can talk to each other, but do not press any other controls while I set up.

<< Once setup complete >>

Driver, remember that your goal is to go as fast as you can without hitting anything, and to follow the green arrows.

[*If drone operator*]

Drone operator, remember to help the drive by communicating information about traffic, or anything else that the driver requests.

The simulation will take approximately 30 minutes. Driver, start when you’re ready and I’ll tell you when to stop.

**Supplemental B: Additional Results from Machine Learning Analysis**

Table B.1

*Machine Learning Analysis Predicting Driving and Gaming Experience*

|  | Model | | | | | |
| --- | --- | --- | --- | --- | --- | --- |
|  | Bayesian Ridge | | Random Forest | | Support Vector Machine | |
| Variable | SMAPE | SD | SMAPE | SD | SMAPE | SD |
| **Speed (probe-free periods)** | |  |  |  |  |  |
| Driving years | 46.04 | 32.52 | 42.56 | 30.86 | 43.27 | 29.33 |
| Gaming time | 37.47 | 23.57 | 41.56 | 31.44 | 35.81 | 28.36 |
| **Speed (event probes)** | |  |  |  |  |  |
| Driving years | 36.84 | 27.86 | 40.40 | 28.57 | 38.85 | 28.20 |
| Gaming time | 41.46 | 32.68 | 43.18 | 31.49 | 48.92 | 33.78 |
| **Collisions (probe-free periods)** | | |  |  |  |  |
| Driving years | 40.46 | 30.83 | 40.87 | 29.39 | 39.59 | 31.20 |
| Gaming time | 36.57 | 30.65 | 41.07 | 32.04 | 41.36 | 29.53 |
| **Collisions (event probes)** | | |  |  |  |  |
| Driving years | 40.34 | 29.32 | 42.46 | 27.93 | 35.39 | 29.58 |
| Gaming time | 50.15 | 35.97 | 43.10 | 31.17 | 39.39 | 28.16 |

*Note.* SMAPE = symmetric mean absolute percentage error.
